# Optimization of Extraction Conditions for Determination of Tiliroside in *Tilia* L. Flowers Using an LC-ESI-MS/MS Method

**DOI:** 10.1155/2019/9052425

**Published:** 2019-01-09

**Authors:** Aleksandra Pieczykolan, Wioleta Pietrzak, Renata Nowak, Józefina Pielczyk, Karolina Łamacz

**Affiliations:** Department of Pharmaceutical Botany, Medical University of Lublin, Chodźki 1 Str., 20-093 Lublin, Poland

## Abstract

Tiliroside exhibits a wide spectrum of effects on the human body; considering expensive synthesis of tiliroside, linden trees seem to be a good source of this compound. For the first time, 46 various extraction methods were developed to receive tiliroside from *Tilia* L., including ultrasound-assisted extraction, maceration, maceration with stirring, accelerated solvent extraction, and extraction under reflux. The effects of extraction techniques, solvents, additives, and temperature on the content of tiliroside were studied using analytical and statistical methods. A new, rapid, simple, sensitive, and selective liquid chromatography-electrospray ionization-tandem mass spectrometry (LC-ESI-MS/MS) method was developed to determine the content of tiliroside in *Tilia* L. flowers. The LC-ESI-MS/MS analysis demonstrated the highest content of tiliroside in *Tilia* L. flowers obtained using accelerated solvent extraction (ASE) where 70% ethanol with addition of 1% acetic acid was used as a solvent (7.400 ± 0.019 mg of tiliroside per g dry extract).The results showed that the extracts of *Tiliae* inflorescentia contained large amounts of tiliroside; therefore, they are good sources of this compound. Moreover, ASE was found to be superior to other extraction techniques due to its high efficiency as well as considerable saving of time and solvent.

## 1. Introduction

**Figure 1:**
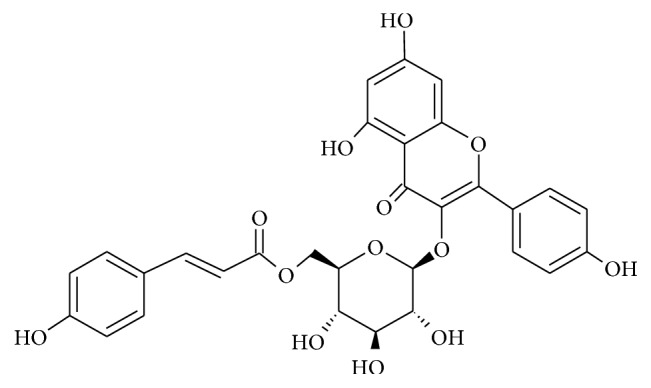
Structure of tiliroside (using the ACD/Chemsketch program, version 12.5 (Advanced Chemistry Development, Inc.)).

Kaempferol–3-O-*β*-D-(6″-*p*-coumaroyl)-glucopyranoside (tiliroside) was isolated for the first time from the leaves of *Tilia argentea* Desf. ex DC by Hörhammer et al. in 1961 [1]. It is a glycosidic flavonoid ester (Figure 1) commonly found in nature in herbal plants and in medicines [2]. According to other literature reports, tiliroside is present in several plant species belonging to different botanical families, e.g., Araliaceae [2], Asteraceae [3], Euphorbiaceace [4], Lamiaceae [5], Malvaceae [5], and Rosaceae [6].

Tiliroside exhibits a wide spectrum of effects on the human body. The plant materials containing tiliroside show antithrombotic [7], anticoagulant [8], hepatoprotective [9], antimicrobial [10], anti-inflammatory [11], and antiproliferative activities [12]. Several in vivo and in vitro studies have indicated its anticancer activity against small-cell lung cancer A549 cells [13], prostate carcinoma PC-3M [14], breast cancer MCF-7 and T47D [12], and leukaemia cells lines [15]. Moreover, according to the most recent findings, tiliroside significantly inhibits body weight gain and accumulation of adipose tissue in mice and decreases the concentration of fatty acids and triglycerides in blood [16, 17].

Due to its antioxidant and anti-inflammatory properties, tiliroside has been used in the cosmetic industry. One of the available studies has shown that tiliroside does not show high activity against free radicals but may be useful as a UV B blocker [18]. According to some other studies, tiliroside is a potent tyrosinase inhibitor and might be important as a skin-whitening agent and a pigmentation medicine [19]. The latest research results have demonstrated potential anti-ageing activity of tiliroside; moreover, the compound was found to exert no toxicity in normal human fibroblasts, to increase proteasome activities and to retard cellular senescence [20].

In combination with sorbitol, tiliroside was used by Merck in the production of a new cosmetic, RonaCare® Tiliroside. According to the manufacturer, the product is mainly intended for dry skin care, shows anti-inflammatory properties, and shields the skin from aching and itching. The above was confirmed by Carola et al. [21] who studied UV-induced experimental stress reaction of the skin. The development of erythema was examined after the application of the respective test substances at the measuring time periods prior to treatment and after different time points in comparison with both a negative and a positive control. RonaCare® Tiliroside was found to have calming, soothing, and anti-inflammatory properties.

*Tilia* L. is commonly found in the temperate climate of Poland. Since the synthesis of tiliroside is expensive, linden trees are a very good source of this compound. Considering an increasing demand for tiliroside and benefits of the research on this compound, in particular regarding its anti-inflammatory effects and possible wider use in the cosmetic industry, our study was designed to find the most efficient method of extraction of this compound.

## 2. Materials and Methods

### 2.1. Plant Material

*Tiliae* inflorescentia (*Tiliae* flos) was purchased from the herb company of Kawon (Gostyń, Poland). According to the information provided by the manufacturer, the plant material was dried in the air and powdered in accordance with the requirements of the Polish Pharmacopoeia VI.

Raw material details are shown in Table 1.

### 2.2. Extraction Methods

Portions of powdered plant material were extracted using various extraction methods, i.e., maceration, maceration with stirring, extraction under reflux, ultrasound-assisted extraction, and accelerated solvent extraction.

Extracts were marked with symbols denoting the individual extraction methods: MAC: maceration, MACst: maceration with stirring, UR: extraction under reflux, ASE: accelerated solvent extraction, UAE: ultrasound-assisted extraction), and the concentration and symbols of solvents are M: methanol, E: ethanol, W: water, DE: diethyl ether, CH: chloroform); additionally the extraction temperatures were provided in brackets (rt: room temperature, 80: 80°C, etc.). Moreover, the influence of addition of acetic acid (AA) and diethyl ether (DE) was studied, and the samples were marked with symbols denoting the compound and its concentration, e.g., MACst-E70% + AA0.1% means the extract obtained by maceration with stirring using 70% ethanol and addition of 0.1% acetic acid.

In the case of maceration, plant material (10 g) was flooded with 100 mL of 70% ethanol, stirred, and put aside for 7 days at room temperature.

Maceration with stirring (10 g of plant material) was conducted at room temperature with the following eluents: water, methanol, and ethanol (50%, 70%, and 100%), 70% and 100% ethanol with addition of acetic acid (0.5, 1, 2, 5, and 10%), 100% ethanol with addition of acetic acid (0.5 and 1%), and 70% ethanol with addition of diethyl ether (5 and 10%). This extraction lasted 2 days, while stirring for 6 hours per day.

The extraction with a boiling solvent under reflux (10 g of plant material) was carried out with 100 mL of 70% ethanol 2 times for 1 hour each (UR-E70%) in duplicate.

One extract was soaked with 9 portions of 100 ml of diethyl ether. Each time, the ether fraction was collected, pooled, evaporated to dryness under vacuum, and lyophilized (UR-DE).

Moreover, the influence of the addition of acetic acid (1%AA) was studied. In this case, 10 g of the plant material was flooded with 100 mL of 70% ethanol with addition of 1% acetic acid. Extraction with a boiling solvent under reflux was carried out 2 times for 1 hour each.

Accelerated solvent extractions (3 times for 10 minutes each) were conducted in the ASE 150 system from Dionex Corporation (Sunnyvale, CA, USA) at 80°C with different methanol and ethanol concentrations (50%, 70%, and 100%) and chloroform (100%).

Moreover, the influence of addition of acetic acid (0.1; 0.5; 1; and 5%) was evaluated.

In the ultrasound-assisted extraction, the plant powder (2 g) was extracted twice (15 minutes each time) with 20 ml of methanol and ethanol (50%, 70%, and 100%). Extractions were performed at room temperature (rt) and at 80°C. The influence of addition of acetic acid (0.5 and 1%) was studied.

In all cases, the extracts obtained were filtered, evaporated to dryness under vacuum, and lyophilized in the Free Zone 1 apparatus (Labconco, Kansas City, KS, USA). The residue was weighed and redissolved in the same solvent as the one used for extraction to obtain stock solutions at suitable concentrations.

All samples were prepared in triplicate.

### 2.3. Total Phenolic and Total Flavonoid Contents

The total phenolic content (TPC) and total flavonoid content (TFC) were determined using 96-well transparent microplates (Nunclon. Nunc. Roskilde, Denmark) and an Infinite Pro 200F microplate reader (Tecan Group Ltd., Männedorf, Switzerland).

Analysis of TPC was carried out using the Folin–Ciocalteu phenol reagent according to the method described by Olech with some modifications [22]. Absorbance was read at 680 nm after 20-minute incubation. TPC was determined using a standard curve prepared for gallic acid. The results were expressed as mg of gallic acid per 1 g of dry weight of the plant material (GAE: gallic acid equivalent).

The total flavonoid content (TFC) was determined according to the modified Lamaison and Carret method [23]. Absorbance was measured at 430 nm after a 30-minute incubation against a blank solution containing methanol instead of the test sample. The results were expressed as mg quercetin (Q) per 1 g of dry extract.

### 2.4. Antiradical Activity Analysis

The antioxidant assay was determined by the DPPH˙ (2,2-diphenyl-1-picrylhydrazyl) method with some modifications [22, 24]. Absorbance was measured at 517 nm using the Infinite Pro 200F microplate reader (Tecan Group Ltd., Männedorf, Switzerland) after 30-minute incubation. The inhibition curves were prepared and IC_50_ values, defined as the amount of antioxidant necessary to decrease the initial DPPH˙ concentration by 50%, were determined. The results were expressed as mg dry extract per 1 mg DPPH˙.

The antiradical activity was assessed using an improved ABTS^+^˙ decolourization assay with modifications [25] for application on the Elisa microplate reader for the first time.

ABTS was dissolved in water to 8.7 mM concentration. ABTS radical cation (ABTS˙^+^) was produced by allowing 1.25 ml of ABTS stock solution to react with 5 ml of potassium persulfate (12.2 mM) and allowing the mixture to stand in the dark at room temperature for 12–18 h before use. The ABTS˙^+^ solution was diluted with methanol (1 : 100).

To determine IC_50_ of samples, the technique with 96-well microplates was used. Aliquots of 180 *μ*l of ABTS^+^˙ solution were mixed with 20 *μ*l of the samples diluted to various concentrations in 96-well microplates. Absorbance was measured after 6-minute incubation at 734 nm using the Infinite Pro 200F microplate reader (Tecan Group Ltd., Männedorf, Switzerland). The extract ability to quench the ABTS˙ free radical was determined using the following equation:

(1) $$\begin{array}{c} \text{scavening }\%=\left[\frac{A_{\text{C}}-A_{\text{A}}}{A_{\text{C}}}\right]\times 100, \end{array}$$

where *A*_C_ is the absorbance of control and *A*_A_ is the absorbance of the sample.

The results of antioxidant activity were expressed as Trolox equivalent antioxidant capacity (TEAC) (mM of Trolox per g sample) based on their IC_50_ values.

### 2.5. LC-ESI-MS/MS Analysis of Tiliroside

#### 2.5.1. Chromatographic Conditions and Apparatus

Tiliroside was determined by a newly developed, simple, and rapid method using liquid chromatography-electrospray ionization-tandem mass spectrometry (LC-ESI-MS/MS).

For this, an Agilent 1200 Series HPLC system (Agilent Technologies, USA) equipped with a binary gradient solvent pump, a degasser, an autosampler, and a column oven connected to a 3200 QTRAP Mass spectrometer (AB Sciex, USA) was used.

The contents of tiliroside were determined. The compound was separated at 25°C on the Zorbax SB-C18 column (2.1 × 50 mm, 1.8-*μ*m particle size; Agilent Technologies, USA). The injection volume was 5 *μ*l. Gradient elution was applied using water containing 0.1% HCOOH (A) and acetonitrile (B). The flow rate was 200 *μ*l/min, and the gradient was as follows: 0–0.5 min 10% B; 1–1.5 min 50% B; 2.5–3.5 min 80% B; 4–5,5 min 70% B; 5–8 min 90% B [26].

#### 2.5.2. Optimization of Parameters for Quantitative Analysis

The 3200 QTRAP MS/MS system was operated using an electrospray ion source in the negative mode. The optimal mass spectrometer parameters were determined experimentally and were as follows: curtain gas 30 psi, capillary temperature 500°C, nebulizer gas 30 psi, and negative ionization mode source voltage −4000 V.

Nitrogen was used as a curtain and collision gas. The data were acquired and processed using Analyst 1.5 software from AB Sciex, USA.

Multiple reaction monitoring (MRM) was used for quantitative analysis of tiliroside. The compound was identified by comparing the retention time and *m*/*z* values obtained by MS and MS2 with the mass spectra of the corresponding standard tested under the same conditions. The calibration curve obtained in the MRM mode was used for analyte quantification. The identified tiliroside was quantified based on its peak area and comparison with a calibration curve for the corresponding standard [26]. The linearity range for the calibration curve was specified. The limit of detection (LOD) and quantification (LOQ) for tiliroside were determined. Tables 2 and 3 show the optimized parameters for quantification of tiliroside.

#### 2.5.3. Intraday and Interday Precisions and Accuracy Data

Validation of the method for precision was measured by using a standard solution containing tiliroside concentrations covering the entire calibration range.

The method was validated for instrumental precision, intraday precision, and interday precision. The instrumental precision was tested by repeated analysis (*n*=10) of a tiliroside standard solution (100 ng/mL) on the same day (Table 4).

The intraday data reflect the precision of the method under the same conditions within one day. Intraday precision was obtained by analyzing six replicates of three tiliroside standard solution concentrations (50, 100, and 500 ng/mL) (Table 5).

The interday precision was verified by repeating the above procedure at three different days within the period of one week (Table 6).

#### 2.5.4. Statistical Analysis

Results were expressed as mean ± standard deviation (SD) of three replications for each extract tested. Moreover, the relative standard deviation (RSD) for instrumental, interday, and intraday precision was determined.

Calculations, spread chart 3W, surface chart 3W, and median graph were performed in STATISTICA 10.0 (StatSoft).

## 3. Results and Discussion

In our study, the effects of a particular extraction technique, solvent, additives, and temperature on the tiliroside content were evaluated. For this purpose, 46 different *Tiliae* inflorescentia extracts were investigated. Classical (maceration, maceration with intensive stirring, and extraction under reflux) and modern (ultrasound-assisted extraction and accelerated solvent extraction) extraction techniques were used.

Tiliroside, as mentioned earlier, possesses anti-inflammatory, antioxidant, anticarcinogenic, cytochrome P450 inhibitory, and hepatoprotective activities. Recently, its anti-diabetic effects have also been reported [17]. Moreover, the biological activities of the natural ingredient tiliroside were investigated for cosmetic application. Tiliroside is a potent ingredient for normal and sensitive skin and offers a multitude of claim opportunities for cosmetics, exhibiting calming, soothing, and anti-inflammatory properties [21]. Due to expensive synthesis of tiliroside, linden trees are a very good source of this compound. Lime trees, commonly found in temperate climates, can be a potential rich source of tiliroside, mostly present in Tiliae inflorescences, which are easily accessible and widely used for medicinal and cosmetic purposes.

Extraction of the plant material is the first and very important step in the analytical process of various studies dealing with plant secondary metabolites [27, 28]. Generally, conventional extraction methods depend mainly on the type of solvent applied for isolation and extraction time, while modern extraction techniques depend also on many different factors characteristic of each method used. Modern extraction techniques are superior to traditional methods in terms of reduction in organic solvent amount, extraction time, and minimizing sample degradation. However, numerous parameters have to be considered in order to select the optimal extraction technique, for instance, the extraction plan and its application, the time needed for extraction, the cost and availability of instruments, and the quantity of sample required for extraction [29, 30].

To date, the extraction process for the highest content of tiliroside from *Tilia* L. flowers has not been optimized. Different extraction methods, extraction procedures, and solvents have been reported for extracting polyphenols (including tiliroside) from *Tilia* L. and determined using the HPLC method [5, 30–32]. This study is the first to optimize the extraction of tiliroside from *Tiliae* inflorescentia using the LC-ESI-MS/MS method.

The extractive yields vary among different solvents, methods, and conditions of extraction.  Table 7 shows the results of extraction yields (calculated as percentage of dry extract obtained from 1 g of raw material) and contents of tiliroside. The extraction efficiency varied from 1.5% for ASE with 100% chloroform (ASE-CH100% (50)) to 27.8% for ASE with 70% ethanol with addition of 5% acetic acid at 80°C (ASE-E70% (80) + AA5%). Dynamic extraction techniques, such as accelerated solvent extraction (ASE) and extraction under reflux (UR), were found to yield higher efficiencies than other extraction techniques. Moreover, in each extraction method used, the addition of acetic acid increased the extraction efficiency.

The liquid chromatography-electrospray ionization-tandem mass spectrometry method has many advantages. It is a simple, rapid, reliable, and effective analytical tool [33]. A new method for determination of tiliroside by LC-ESI-MS/MS was developed and applied for the first time of tiliroside. This step was a very important aspect of our study.

First, the method was validated. The correlation coefficient for tiliroside over the concentration range of 2–500 ng·mL^−1^ was 0.9995. The limit of detection (LOD) and the limit of quantitation (LOQ) at a signal-to-noise ratio of three and ten (S/N = 3 and 10) for tiliroside were 0.5 and 2 ng·mL^−1^, respectively (Table 5).

Instrumental precision was tested by repeated analysis (*n*=10) of a tiliroside standard solution (100 ng·mL^−1^) on the same day. The relative standard deviation (RSD) for instrumental precision was 2% (Table 4).

The intraday and interday precisions for tiliroside standard are summarized in Tables 5 and 6. The percentage RSD of intraday and interday precisions of tiliroside was 0.3–2.1%. The acceptance criteria are not more than 15% deviation from the nominal value for precision and accuracy. It is desirable that these tolerances be provided both for intraday and interday experiments [34]. The validation results indicated that the method was precise.

Table 7 shows the tiliroside content according to the method and extraction conditions used. Comparing maceration and maceration with stirring, more tiliroside was detected in the extracts obtained with the latter (1.717 ± 0.005 mg·g^−1^ dry extract for MACst-E70% and 1.530 ± 0.001 mg·g^−1^ dry extract for MAC-E70%). Mixing was found as an important factor affecting this extraction type, as it increased the amount of tiliroside eluted from the plant material. Moreover, higher extraction of tiliroside was obtained for extracts using the solvent with addition of acetic acid (AA) or diethyl ether (DE).

**Figure 2:**
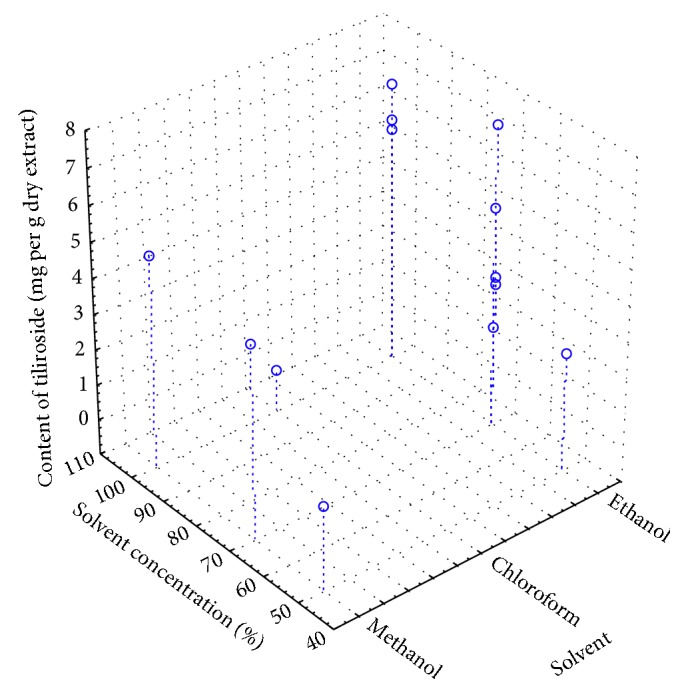
Spread chart 3W of the content of tiliroside vs. type of solvent and solvent concentration for ultrasound-assisted extraction (UAE).

In the case of ultrasound-assisted extraction (Figure 2), the content of tiliroside in extracts from *Tilia* L. was greatly influenced by the solvent concentration in water. The higher the solvent concentration, the higher the extraction. The highest amounts of tiliroside were obtained for UAE-M100% (rt) (4.281 ± 0.084 mg·g^−1^ dry extract) and UAE-E100% (rt) (3.291 ± 0.059 mg ·g^−1^ dry extract) extracts.

**Figure 3:**
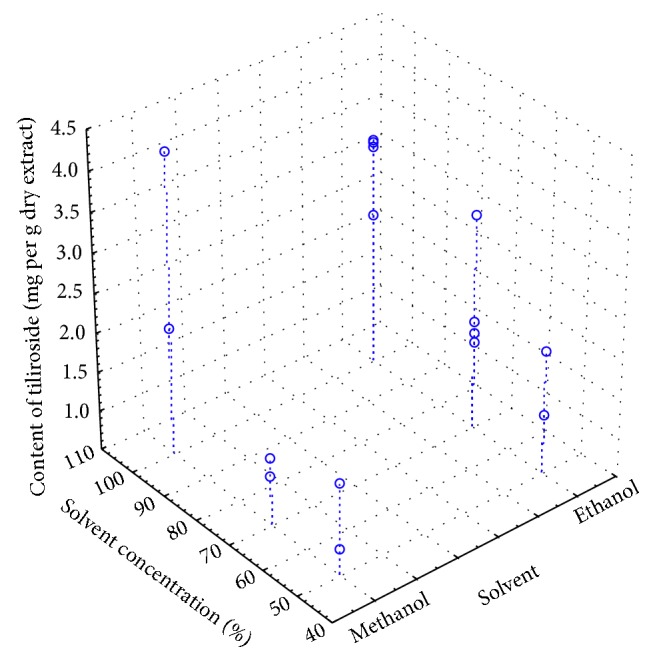
Spread chart 3W of the content of tiliroside vs. type of solvent and solvent concentration for accelerated solvent extraction (ASE).

Similar dependencies were observed for accelerated solvent extraction (Figure 3). The extraction of tiliroside increased with an increase in solvent concentration. However, higher amount of tiliroside was obtained for ethanol extracts.

Noteworthy, the choice of an appropriate solvent is of utmost importance along with application of a compatible extraction method [30]. Since ethanol is nontoxic and can be mixed with water in different ratios, it was chosen for extraction of tiliroside from *Tiliae* inflorescence.

**Figure 4:**
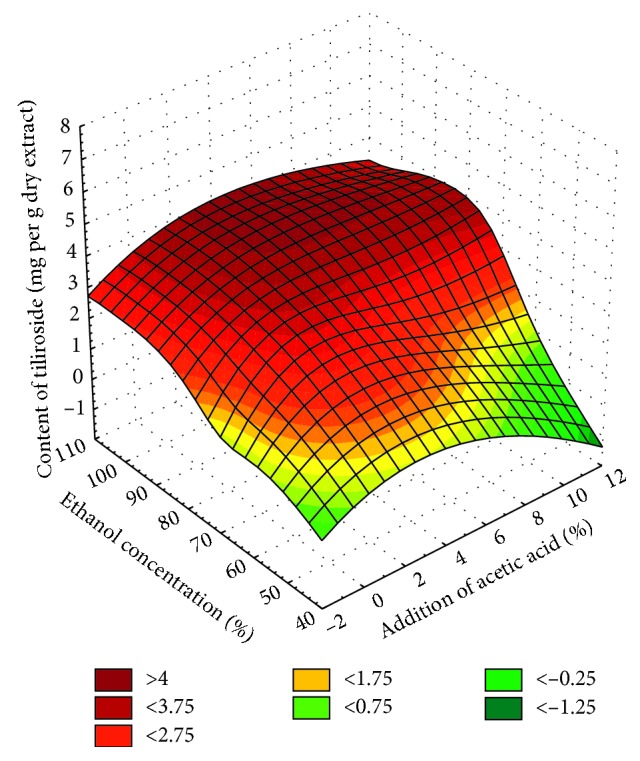
Surface chart 3W of the content of tiliroside vs. addition of acetic acid and ethanol concentration for all extraction methods (smoothing of smallest squares weighted by distance).

Moreover, the analysis of all ethanol extracts (Figure 4) indicated that the extraction of tiliroside increased with an increase in ethanol concentration and with addition of acetic acid (optimum amount of acetic acid, about 1–2%). However, it should be noted that the addition of acetic acid to ethanol extracts exceeding 1-2% did not increase the extraction of tiliroside.

**Figure 5:**
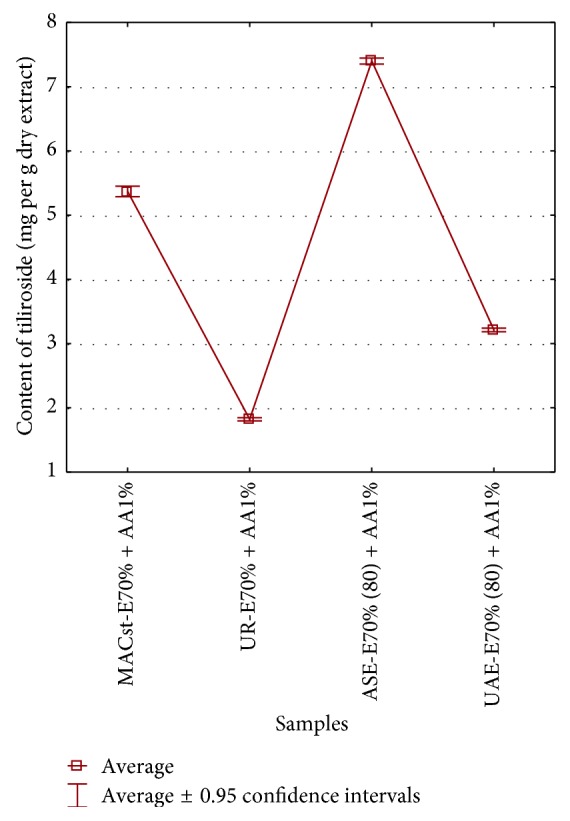
Median graph of the content of tiliroside vs. different extraction methods.

70% ethanol with addition of 1% acetic acid was selected as the best solvent for extraction of tiliroside.  Figure 5 shows the comparison of tiliroside contents vs. extraction methods using 70% ethanol with addition of 1% acetic acid as a solvent. The results show that the best method for using this solvent was ASE (7.400 ± 0.019 mg of tiliroside per g dry extract).

Moreover, for MACst-E70% + AA1%, ASE-E70% (80) + AA1%, UAE-E70% (80) + AA1%, and UR-E70% + AA1% samples, spectrophotometric determination of total phenolic content (TPC) and total flavonoid content (TFC) as well as antioxidant activity tests were performed (Table 8).

The antiradical activity was assayed using a DPPH^•^ method with some modifications [22, 24] and an improved ABTS^+^˙decolourization assay [25] with modifications for application on an Elisa microplate reader, described for the first time. All the extracts exhibited high antioxidant activity. TFC varied from 14.05 for UR-E70% + AA1% samples to 23.70 mg·Q·g^−1^ of dry extract for MACst-E70% + AA1% samples while TPC varied from 181.39 to 296.96 mg·GA·g^−1^ of dry extract for ASE-E70% (80) + AA1% samples.

The tested extracts also contained a high amount of polyphenols and flavonoids and had high antioxidant activity (Table 8).

## 4. Conclusions

In conclusion, the results of this study showed that the extracts of *Tiliae* inflorescentia contained large amounts of tiliroside; therefore, *Tiliae* inflorescentia are good sources of this compound.

Our study presented a newly developed method for determination of tiliroside using simple, rapid, and reliable liquid chromatography-electrospray ionization-tandem mass spectrometry, appropriate to industrial applications.

The effective methods to receive tiliroside from *Tilia* L. flowers have been developed for the first time. The ASE method using 70% ethanol with addition of 1% acetic acid as a solvent proved to be the best way to extract tiliroside from *Tilia* L. flowers. Additionally, compared to classical extraction methods, UAE and ASE provided high extraction yields, requiring short time frames and less labour.

Accelerated solvent extraction is an appropriate method for analytical application. However, due to economic conditions, classical maceration with the same solvents also giving a very active extract with high amounts of tiliroside and other phenolic compounds can be recommended for industrial application.

## Figures and Tables

**Table 1:** Raw material details.

Plant material	Systematic affiliation	Harvest time	Origin
*Tiliae* inflorescentia (*Tiliae* flos)	*Tilia cordata* Mill. and/or *Tilia platyphyllos* L. (Tiliaceae)	June-July 2015	KAWON company

**Table 2:** Summary of optimized parameters for quantitative analysis of tiliroside.

Compound	Retention time (min)	Q1 (*m*/*z*)	Q3 (*m*/*z*)	DP^a^ (V)	EP^b^ (V)	CEP^c^ (V)	CE^d^ (eV)	CXP^e^ (V)
Tiliroside	6.17	302.7	124.9	−45	−3.5	−18	−26	0
			284.8	−45	−3.5	−18	−14	−4

^a^DP: declustering potential; ^b^EP: entrance potential; ^c^CEP: cell entrance potential; ^d^CE: collision energy; ^e^CXP: collision cell exit potential.

**Table 3:** Analytical parameters of LC-MS/MS quantitative method.

Compound	LOD (ng/mL)	LOQ (ng/mL)	*R* ^2^	Linearity range (ng/mL)
Tiliroside	0.5	2	0.9995	2–500

**Table 4:** Instrumental precision.

Nominal concentration (ng/mL)	Measured concentration (*n*=10)	
	Mean ± SD	% RSD
100	104.00 ± 2.32	2.2

**Table 5:** Intraday precision data.

Nominal concentration (ng/mL)	Measured concentration (*n*=6)	
	Mean ± SD	% RSD
50	51.06 ± 1.04	2.0
100	103.00 ± 1.90	1.8
500	499.80 ± 4.79	1.0

**Table 6:** Interday precision data.

Nominal concentration (ng/mL)	Measured concentration (*n*=18)	
	Mean ± SD	% RSD
50	51.01 ± 1.06	2.1
100	103.12 ± 2.90	1.0
500	499.97 ± 4.80	0.3

**Table 7:** Tiliroside contents in extracts from *Tilia L* (±SD, *n*=3).

Samples	Efficiency of extraction (%)^a^	Content of tiliroside (mg per g dry extracts)
MAC-E70%	13.7	1.530 ± 0.001
MACst-H	17.4	0.142 ± 0.005
MACst-E50%	14.6	1.105 ± 0.010
MACst-E70%	17.4	1.717 ± 0.005
MACst-E100%	5.3	1.289 ± 0.002
MACst-E70% + AA0.5%	18.5	5.010 ± 0.024
MACst-E70% + AA1%	18.4	5.370 ± 0.033
MACst-E70% + AA2%	20.1	2.870 ± 0.011
MACst-E70% + AA5%	21.1	2.540 ± 0.010
MACst-E70% + AA10%	19.5	2.990 ± 0.009
MACst-E70% + DE5%	15.4	3.900 ± 0.013
MACst-E70% + DE10%	13.2	3.560 ± 0.012
MACst-E100% + AA0.5%	6	2.130 ± 0.008
MACst-E100% + AA1%	6	2.160 ± 0.006
ASE-M100% (80)	14.8	4.980 ± 0.037
ASE-M70% (80)	22.3	4.440 ± 0.028
ASE-M50% (80)	22.0	1.371 ± 0.013
ASE-CH100% (50)	1.5	0.193 ± 0.001
ASE-E100% (80)	3.3	6.810 ± 0.014
ASE-E70% (80)	19.6	2.980 ± 0.007
ASE-E50% (80)	8.8	2.320 ± 0.012
ASE-E70% (80) + AA0.1%	23.0	3.200 ± 0.013
ASE-E70% (80) + AA0.5%	18.9	5.160 ± 0.017
ASE-E70% (80) + AA1%	26.4	7.400 ± 0.019
ASE-E70% (80) + AA5%	27.8	1.802 ± 0.004
ASE-E100% (80) + AA0.5%	18.9	5.850 ± 0.012
ASE-E100% (80) + AA1%	10.8	5.560 ± 0.015
UR-E70%	23.6	1.225 ± 0.006
UR-DE	5.6	9.842 ± 0.028
UR-E70% + AA1%	18.1	1.821 ± 0.010
UAE-M100% (rt)	3.6	4.281 ± 0.084
UAE-M70% (rt)	5.1	1.358 ± 0.016
UAE-M50% (rt)	6.6	0.829 ± 0.004
UAE-E100% (rt)	2.5	3.291 ± 0.059
UAE-E70% (rt)	4.0	1.728 ± 0.028
UAE-E50% (rt)	4.1	1.244 ± 0.019
UAE-M100% (80)	9.4	2.116 ± 0.038
UAE-M70% (80)	9.2	1.120 ± 0.019
UAE-M50% (80)	5.4	1.650 ± 0.054
UAE-E100% (80)	7	2.406 ± 0.001
UAE-E70% (80)	7.5	1.607 ± 0.007
UAE-E50% (80)	5.8	2.072 ± 0.015
UAE-E70% (80) + AA0.5%	8.4	1.848 ± 0.004
UAE-E70% (80) + AA1%	6.6	3.212 ± 0.021
UAE-E100% (80) + AA0.5%	2.8	3.322 ± 0.012
UAE-E100% (80) + AA1%	4	3.368 ± 0.057

^a^Calculated as percentage of dry extract obtained from 1 g of raw material.

**Table 8:** Total phenolic content (TPC), total flavonoid content (TFC), antioxidant activity by the DPPH˙ method, and free radical scavenging ability (ABTS^+^˙) in different extracts (70% ethanol with addition of 1% acetic acid) from *Tilia* L. flowers.

Sample	TPC (mg·GA·g^−1^ of dry extract)	TFC (mg·Q·g^−1^ of dry extract)	TEAC (mM·Trolox·g^−1^ dry extract)^*∗*^	IC_50_ (mg·mg^−1^·DPPH˙)
MACst-E70% + AA1%	239.09 ± 15.66	23.70 ± 0.89	1.06 ± 0.03	0.25 ± 0.01
ASE-E70% (80) + AA1%	296.96 ± 15.66	16.89 ± 0.86	1.54 ± 0.05	0.30 ± 0.02
UAE-E70% (80) + AA1%	238.69 ± 15.46	20.58 ± 0.08	1.54 ± 0.02	0.25 ± 0.01
UR-E70% + AA1%	181.39 ± 2.62	14.05 ± 0.71	1.17 ± 0.01	0.34 ± 0.01

## Data Availability

The data used to support the findings of this study are included within the article.
